# Rheumatoid arthritis and osteoarthritis patients demonstrate comparable rates of major adverse cardiovascular events: a single-centre retrospective cohort study

**DOI:** 10.1093/rap/rkaf145

**Published:** 2025-12-13

**Authors:** Chung M A Lin, Hadeel M Tabaqchali, Stephen White, Arthur G Pratt, John D Isaacs, Faye A H Cooles

**Affiliations:** Translational and Clinical Research Institute, Newcastle University, Newcastle-upon-Tyne, UK; Rheumatology Department, Musculoskeletal Services, Newcastle upon Tyne Hospitals NHS Foundation Trust, Newcastle-upon-Tyne, UK; NIHR Newcastle Biomedical Research Centre (BRC) based at the, Newcastle Hospitals NHS Foundation Trust and Newcastle University, Newcastle-upon-Tyne, UK; The Medical School, Newcastle University, Newcastle-upon-Tyne, UK; Faculty of Medical Sciences, Biosciences Institute, International Centre for Life, Newcastle University, Newcastle-upon-Tyne, UK; Translational and Clinical Research Institute, Newcastle University, Newcastle-upon-Tyne, UK; Rheumatology Department, Musculoskeletal Services, Newcastle upon Tyne Hospitals NHS Foundation Trust, Newcastle-upon-Tyne, UK; NIHR Newcastle Biomedical Research Centre (BRC) based at the, Newcastle Hospitals NHS Foundation Trust and Newcastle University, Newcastle-upon-Tyne, UK; Translational and Clinical Research Institute, Newcastle University, Newcastle-upon-Tyne, UK; Rheumatology Department, Musculoskeletal Services, Newcastle upon Tyne Hospitals NHS Foundation Trust, Newcastle-upon-Tyne, UK; NIHR Newcastle Biomedical Research Centre (BRC) based at the, Newcastle Hospitals NHS Foundation Trust and Newcastle University, Newcastle-upon-Tyne, UK; Translational and Clinical Research Institute, Newcastle University, Newcastle-upon-Tyne, UK; Rheumatology Department, Musculoskeletal Services, Newcastle upon Tyne Hospitals NHS Foundation Trust, Newcastle-upon-Tyne, UK; NIHR Newcastle Biomedical Research Centre (BRC) based at the, Newcastle Hospitals NHS Foundation Trust and Newcastle University, Newcastle-upon-Tyne, UK

Dear Editor, RA was historically associated with an increased incidence of major adverse cardiovascular and cerebrovascular events (MACCE) [1, 2]. However, more recent literature has suggested narrowing of the MACCE incidence gap between RA and the general population, with a reduced incidence in MACCE seen in patients diagnosed after the year 2000 [3]. A progressive shift in clinical practice towards earlier rheumatology reviews, earlier use of biologics and tighter disease control has resulted in a new ‘modern RA’ cohort [3, 4].

OA has also been linked to increased MACCE incidence when compared with population controls [5]. Yet historical head-to-head comparisons between RA and OA in the early 2000s still demonstrated a 2-fold increase in MACCE incidence in RA *vs* OA, even after adjusting for conventional cardiovascular risk factors [6]. In RA, other disease-specific factors are likely additionally implicated in MACCE, e.g. pro-inflammatory cytokines [6]. The adoption of a ‘treat-to-target’ approach with DMARDs in RA over recent decades has led to a more rapid and sustained reduction in inflammation with resulting decreased circulating inflammatory cytokines and improved overall disease control [7]. Given this current therapeutic landscape, we sought to explore MACCE incidence in ‘modern’ RA and OA populations and identify if rapid inflammatory disease control in the first 6 months of RA diagnosis influenced subsequent MACCE risk.

Individuals attending the North-East Early Arthritis Clinic (NEAC) receiving a new clinical diagnosis of RA or OA between December 2011 to November 2021 were recruited as part of a single-centre retrospective study. MACCE occurring after OA/RA diagnosis until 30 April 2024 were recorded based on hospital coding via Newcastle-upon-Tyne Hospitals NHS Foundation Trust electronic health records. Time to MACCE (in years) was calculated from the date of first rheumatology clinic review to the approximate date of MACCE diagnosis in the patient records. MACCE codes included stroke, transient ischaemic attack (TIA), myocardial infarction (MI) and a new diagnosis of ischaemic heart disease (IHD). We also collected age, sex, baseline BMI, blood pressure, comorbidities, smoking and alcohol use, family history of IHD, musculoskeletal symptom duration and level of deprivation, as determined by an index score generated from the English indices of deprivation 2019 calculator. The Charlson comorbidity index (CCI) was also calculated, which includes measurement of conventional MACCE risk factors. For RA patients, serostatus (determined by RF and/or ACPA positivity), baseline disease activity scores [28-joint DAS with ESR (DAS28-ESR)] and 6-month EULAR clinical responses to initial DMARDs were also collected. Ethical approval was obtained from the North-East–Newcastle & North Tyneside 2 Research Ethics Committee (REC: 12/NE/0251). All participants gave written informed consent in accordance with the Declaration of Helsinki.

Patients with a prior history of MACCE were excluded from analyses. Survival and Cox proportional hazards modelling covariates included age, sex, BMI, CCI, hypertension, hyperlipidaemia, deprivation index, smoking and musculoskeletal symptom duration prior to diagnosis. Analyses were censored to account for variable follow-up time. RStudio (Posit, Boston, MA, USA) was used, with significance at *P* < 0.05.

Cohorts included 245 RA and 367 OA patients. RA patients were older at diagnosis [median age 67 years (range 25–95)] than OA patients [median age 64 years (range 27–97)] (*P* = 0.002). Sex ratio (M:F) was ≈1:2 for RA and ≈1:4 for OA (*P* ≤ 0.001) and a higher co-morbidity burden was seen in OA (CCI; *P* = 0.004). The mean BMI was higher (*P* = 0.016) in OA [29.16 kg/m^2^ (range 15.5–59.7)] compared with RA [27.82 kg/m^2^ (range 16.5–47.7)] and smoking rates and deprivation indices were comparable between cohorts (*P* > 0.05). The median musculoskeletal symptom duration pre-diagnosis was longer for OA patients than RA (28.5 *vs* 14 weeks; *P* ≤ 0.001) and the median follow-up time was 7 years (IQR 2–11) for RA and 8 years (IQR 2–12) for OA patients (*P* = 0.035) (Supplementary Table S1, available at *Rheumatology Advances in Practice* online).

The median observed rate estimates for MACCE was 2 events/year in the RA group compared with 3 events/year in the OA subgroup, with a combined median of 4.5 events/year. During follow-up, the MACCE incidence was ≈8 events/1000 person-years in both the OA and RA subgroups, indicating, in these data, no meaningful difference in the event rate between the two diagnoses.

Cox regression analyses confirmed associations of traditional risk factors (smoking and CCI) towards an increased MACCE risk. However, when accounting for population characteristics, including these risk factors, MACCE incidence was comparable between OA and RA (Kaplan–Meier, *P* = 0.91) (Fig. 1A). RA subgroup stratifications also found comparable MACCE risk across groups split by serostatus, EULAR DAS28-ESR clinical response at 6 months (Fig. 1B), disease activity at diagnosis (DAS28-ESR) (Fig. 1C) and musculoskeletal symptom duration pre-diagnosis (<1 year *vs* >1 year) (*P* > 0.05 for all).

**Figure 1:**
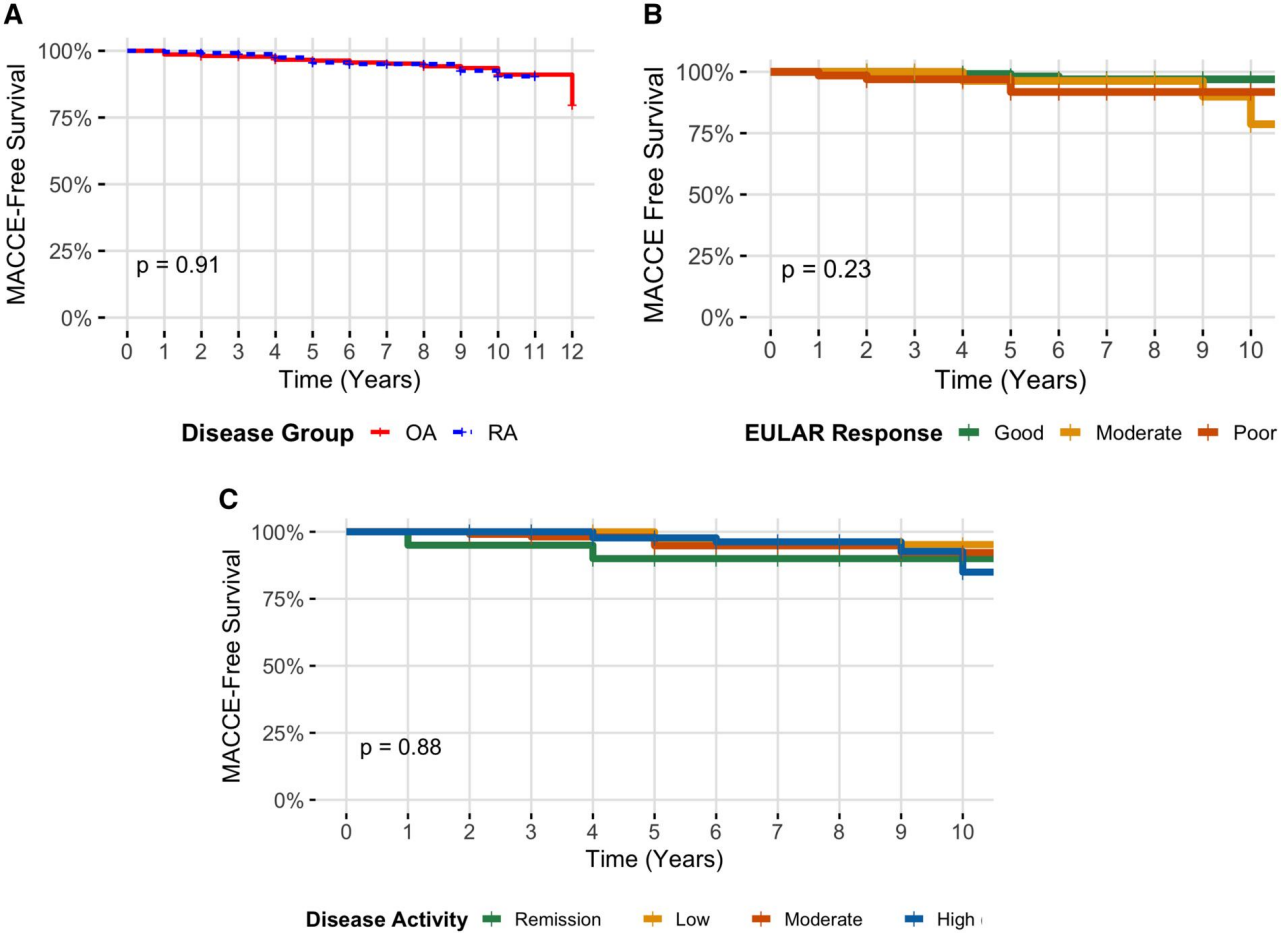
Kaplan–Meier plots demonstrating MACCE-free survival **(A)** between the OA (*n* = 367) and RA (*n* = 245) cohorts; **(B)** within the RA cohort, stratified into three disease response groups determined by 6-month EULAR DAS28-ESR responses (good, *n* = 113; moderate, *n* = 57; poor *n* = 66); **(C)** within the RA cohort, stratified into four disease activity subgroups determined by patient disease activity scores (DAS28-ESR) at baseline (remission, *n* = 20; low, *n* = 23; moderate, *n* = 105; high, *n* = 91)

As our study largely relies on accurate documentation and coding in the patient records, limitations to this study include possible miscoding of clinical events resulting in inaccurate patient numbers, and patient records confined to a single centre, meaning those who have moved away and/or who were lost to follow-up will have limited available data. Furthermore, the number of OA and RA patients who went on to have a MACCE is relatively small despite a long follow-up period, which can result in limited power. Future strategies to overcome these limitations would be to expand the study to multiple centres and recruit larger numbers and over a longer follow-up period.

In summary, data from this large single-centre cohort study demonstrate no difference in MACCE rates between ‘modern’ RA and OA, suggesting a trend towards equalising incidence rates between the two groups. In RA, this apparent reduction in MACCE was independent of early clinical response to DMARDs or baseline disease activity but could be attributed to earlier clinic reviews, longer-term tighter disease control, as well as earlier use of biologics [8]. Cumulatively, these modifications are likely to reduce the level of chronic inflammation historically seen in RA and thus mitigate any resulting adverse cardiovascular effects, as seen in studies assessing biologic responses [8]. Nevertheless, despite these promising data, both modern RA and OA are likely to retain an increased ongoing MACCE risk. As such, proactive primary prevention strategies to reduce longer-term cardiovascular risk and morbidity remain of key importance in these populations.

## Supplementary Material

rkaf145_Supplementary_Data

## Data Availability

The data underlying this article will be shared upon reasonable request to the corresponding author.
