# Supplementary material for: Rheumatoid arthritis and osteoarthritis patients demonstrate comparable rates of major adverse cardiovascular events: a single-centre retrospective cohort study
Source: Rheumatol Adv Pract. 2025 Dec 13;10(1):rkaf145. doi: 10.1093/rap/rkaf145 (PMC12776355; doi:10.1093/rap/rkaf145)
Supplement: rkaf145_Supplementary_Data [file rkaf145_supplementary_data.docx]

**Supplementary Table**

|  | **RA (N=245)** | **OA (N=367)** | **P Value** |
| --- | --- | --- | --- |
| **Age (years): median [range]** | 67 [25-95] | 64 [27-97] | 0.002 |
| **Sex Male:Female** | 79:166 (~1:2) | 67:300 (~1:4) | <0.001 |
| **Smokers (n) (%)** | 25 (10.2%) | 41 (11.1%) | 0.806 |
| **BMI (kg/m2): mean [range]** | 27.82  [16.5-47.7] | 29.16  [15.5-59.7] | 0.016 |
| **Musculoskeletal symptom duration (weeks): median [range]** | 14 [1-105] | 28.5 [0-1200] | <0.001 |
| **Follow-up time (years): median [range]** | 7 [2-11] | 8 [2-12] | 0.035 |
| **Deprivation Index (DI, decile): median [range]** | 4 [1-10] | 4 [1-10] | 0.194 |
| **Comorbidity burden (CCI, points): median [range]** | 4 [2-12] | 3 [0-17] | 0.004 |

**Supplementary Table S1:** Demographics and characteristics across the Rheumatoid Arthritis (RA) and Osteoarthritis (OA) subgroups. CCI; Charlston comorbidity index. Mann Whitney U test for age, DI, CCI, symptom duration and follow-up time. Chi squared test for sex and smoking.
